# Ibn Sina’s (Avicenna) Contributions in the Treatment of Traumatic Injuries

**DOI:** 10.5812/traumamon.4695

**Published:** 2012-07-31

**Authors:** Mohammad Ghannaee Arani, Esmaiel Fakharian, Abolfazl Ardjmand, Hashem Mohammadian, Mahdi Mohammadzadeh, Fahimeh Sarbandi

**Affiliations:** 1 Trauma Research Centre, Kashan University of Medical Sciences, Kashan, IR Iran; 2 Physiology Research Centre, Kashan University of Medical Sciences, Kashan, IR Iran; 3 Department of Health Educations, Kashan University of Medical Sciences, Kashan, IR, Iran

**Keywords:** Avicenna, Wounds and Injuries, Medicine

## Abstract

Modern medicine owes much to the endeavours and contributions made by the ancients that are unfortunately anonymous or even neglected intentionally today. This study was done to give attention to “the ancient golden times”, as the author believes it deserves the nomination, to give credit to the manner our ancient physicians and masters practiced medicine and managed traumas in particular in a way that remains still unrivalled. Undoubtedly such masters as Galen of Pergamon, Hippocrates, Paul of Aegina and Avicenna paved the road for the so-called modern medicine and trauma surgery. Focus of this study is on Ibn Sina or Avicenna as the westerners call him and his methods in handling traumas of any kind and with any severity in the eleventh century based on the teachings handed down to him from the ancients; but he was not a mere imitator. What made him Avicenna was his genius talent in arranging the puzzles in such a way that was not even imagined by the others.

## 1. Introduction

Man’s preoccupation with injuries especially those of the head is not a new one. Even the findings from the Stone Age cave dwellers suggest manipulated interventions in which part of the skull had been chipped away to provide an opening through which the evil spirit could escape (1). With this regard, trepanation has been with man since he understood himself, no matter whether it was operated at the service of religious mythical ceremonies or for healing purposes. Trepanation of the human skull dating to the Neolithic period (about 7000_BC_ to 3000_BC_) has been practiced to remove a piece of calvarium without damaging the underlying blood vessels, meninges, and the brain(1) Della Cook (2000) points out that “trepan” was first used in about 14000 AD to describe a crown saw employed as a surgical instrument. The word we read derives from the Greek *trepanon*, a border (2). By the second century AD, *trepanation* was an established procedure for dealing with skull fracture and its consequences.

The medical treatises attributed to Hippocrates (late fifth century BC) indicate that trepanation was used to relieve the effects of skull fracture and contain descriptions of cylindrical toothed saws. Similar objects were described by the Roman medical writer Celsus (c. 3 AD to 64 AD), who also left instructions on how the instrument should be used. These instructions imply that the instrument had a central pin that could be removed once the saw had begun to penetrate the bone (3). And from Rome we have the account by Celsus (25 _BC_-37 _AD_) of a method of operation which became standard in the surgical books of the Middle Ages. Celsus, whose method differed from that of prehistoric times, advises trepanation for head wounds and gives careful and precise instructions on methodology in his treaties De Medicina, and part of his De Artibus, written between 25 and 35 AD. The foremost physician who employed trepanation in such cases (4).

Medically speaking and as far as injuries are concerned, the term trauma is originally a Greek term meaning hurt or wound. Galen in his book Diseases *And Symptoms* describes the term in his own words “ It is necessary still to speak of the cause of genesis of one further class of disease common to all parts, whether these be *homoiomeric* and entirely simple, or combined. I am accustomed, then, to call this whole class a dissolution of unity, or a destruction of unity, or dissolution of continuity, or however else I would hope the argument will be clear to those hearing it. For we have not received any term concerning this, established by those who have gone before, just as in the case of certain forms of this, when there is dissolution of continuity in bone [they speak of] fracture or caries (*teredon*), and ulcer (*helkos*) or wound (*trauma*) in flesh” (5).

Paul of Aegina’s (625-690 _AD_) surgery reveals certain regressive trends which dominated therapy throughout the Middle Ages. He firmly established the open treatment, particularly in the operative wounds (6).

Many case histories are reported by al-Zahravi (Albucasis) (936-1013) in which he has successfully treated interesting series of unusually severe injuries, often with imaginative and original methods. Guy de Chauliac cites Albucasis close to two hundred times, and as late as the sixteenth century. William Harvey’s teacher, Fabricius of Aquapendente, acknowledged his obligations to three earlier writers, Albucasis, Celsus, and Paul of Aegina (6).

## 2. Ibn Sina, the Prince of Physicians 

Surnamed as the “prince of physicians” Ibn Sina (Bukhara 980-Hamadan 1037) manifested from the most tender age, an extraordinary disposition for the sciences of his time. As soon as arriving at the university of Baghdad to study philosophy and medicine, his talents soon flourished. Avicenna composed numerous works among which “Al-Quanun Fi Al-Tibb (the Canon of Medicine) is the principal one. No author, after Galen, enjoyed such wide and durable authority in the medical world (7). The Canon is divided into five books (daftar) each of which is comprised of treatises (fen) per se. The first book concerned general medical principals, the second with Material Medica. The third and fourth books contain the description and treatment of all the diseases and the last one treats of the composition and preparation of remedies. Like Rhazes, Avicenna was a man with many interests outside of medicine. He left more than 250 books and treatises throughout his fruitful life. Avicenna’s Canon of Medicine has been described as the most studied medical treatise of all time. The Canon brilliantly assimilates and packages the Greek medical wisdom and Islamic medical experience in a logical and well ordered form never written in the field of medicine (8). The Canon was widely read by the Europeans in the Latin translation of Gerard of Cremona made in the twelfth century (9).

## 3. Cases of Trauma in the Canon of Medicine 

To Avicenna, one of the main and initiative cause of diseases in the human body is attributed to the foreign objects, traumas, cold/warm temperature, or cold/warm dishes and drinks (10). Avicenna goes on to put emphasis on the outside factors and injuries such as falls, cuts and traumas in the formation of diseases like deformation and physical weakness which are not due to such inner causes as temperaments and elements (11) . Anatomically speaking, Ibn Sina in Book I of Canon of Medicine deals with the different aspects of the human skeleton where God has thought about strategies to minimize injuries and traumas to the body as much as possible. He specifically describes how the skull bones have been arranged in a way to protect the skull against the injuries and traumas (12).

Elsewhere in the Canon, Avicenna describes the unique structure of the backbone in the prevention of traumas to the back: “Say the backbone in its overall shape is a part of the body which has gained its best shape that is spherical. Because spherical shape is safer than any other one against injuries and traumas, and this is due to this round shape that upper processes are bent downwards and lower processes are bent upwards and all are met in the thoracic vertebrae which is the tenth vertebra “(13).

Managing trauma patients is of much importance depending on the injury severity in which following an efficient triage system has been taken for granted. In this regard, Ibn Sina advises the physician to reveal the pain first if the patient has been affected by an injury or fall (14). Also those injured or fallen from a height are advised for venesection to prevent subsequent swelling (15). This swelling is given quite tremendous attention throughout the Canon. Handling hematomas resulting from injuries especially those made because of ruptures in the soft tissues are considered another vital action guaranteeing the injured from severe consequences such as infection and even limb necrosis. Here, Avicenna emphasizes on evacuating the blood as soon as possible, or else the hematoma prevents the wound from healing and grave outcomes as mentioned above ensue.

In Book VI, in various chapters, Ibn Sina elucidates a wide variety of trauma types with their complications. Beginning with the chapter entitled as Fall and Injury, he believed that from being fallen and injured a number of injuries are likely to occur which result in pain and aches (16).

Ibn Sina goes on to focus on some post-traumatic conditions suggestive of the patient’s status thereafter:

It is expected that if there exists a rupture in the veins of head or liver or spleen, the patient vomits blood or nose bleeding intensively and horribly ensued. It is likely that from being fallen and injured, a man’s stomach is distended; his voice is lost, is affected with rapid successive breaths and is unable to speak. If one had fallen down or injured, for example has hit a wall etc. or some sharp-pointed and pungent object has been put through and penetrated into his body and has been beaten a lot, and he all of a sudden vomits blood and gets diarrhea, you know that his death has arrived. If upon falling, one’s ear is extremely hurt and copious blood flows from his ear, swelling and then his death is certain. If somebody hits the ground from the head and is injured, frequently at the first stage he loses his speech. If this speechlessness lingers to the third day and the patient’s status remains unchanged, the physician must perform enema on him on the third day and wait till the seventh day and must not move the head before that time (16).

Cranial injuries have received excessive attention in the Canon VI, chapter I where Avicenna again focuses on medicating the abscesses, but at the same time, lays emphasis on supervising the fractures primarily if they really exist. According to Ibn Sina, failing to notice the possible fractures beneath the fissure may bring corruption and contribute to intense fever, tremor, destruction of reason, and other symptoms (17) . He also insists that the entire fissure should be opened so that the pus is not retained. He mentions that this curative precaution is applicable not only for the cranial fractures, but also for other parts of the body.

Knowing the exact cause of injury and assessing its dimensions is the best strategy aiding the physician in managing the disease:The point at which one turns away from opinion to accurate fact and then to conviction rests upon the consideration of the cause of the fracture and the assessment of the force of the impinging object, its weight, size, and strength. In this way the true apprehension of the situation will be reached. The accompanying symptoms similarly will sometimes indicate a fracture, that is, loss of vision, voice, and similar ills(18).

Haly also says on signs of skull fractures:”The recognition of any skull fractures arises from the nature of the object which strikes the cranium, its weight and hardness, the force of the blow and from its results, such as impaired vision, loss of speech, or a sudden fall. The examination can also be made when the bone is exposed, especially if the break in the skin is large. If the break or fissure is small you may determine the situation by careful inquiry using an instrument such as a probe. You will recognize the fracture from the sound itself made by the instrument because it will be indicated by a clearly hollow or hoarse sound”(18).

Careful and detailed examination of the patient is of much value before proceeding to the treatment. Ibn Sina believed that sometimes the cranium is ruptured in some part by a contusion and there is not only one fracture but many fractures nevertheless it is not clear there is more than one. Thus, one must open the contused area since sometimes there are more than one fracture but they are not opposite the contused area and not far distant but some clear to the sight because of the contusion and laceration of the skin which is of such a nature and size that one fracture appears while near it are some other hidden fractures. In such cases the physician in consideration of the magnitude of the cause of the fracture should operate and he will discover multiple fractures. Not only does it happen that there may be more than one fracture but one alone which is sufficiently longer and longer than the fissure in the skin. Therefore it is best always to widen the fissure in width and length and thus to avoid errors in managing the condition.

In the case of resorting to surgery and moving the fractured bones of the skull, Avicenna describes a number of instruments for managing the situation. Some are to perforate the bone, some for pulling out the bones, and some for elevating depressed bones. All the tools have different shapes due to the diversity of human heads and situations ahead of the physician. Thus, the prudent physician is always expected to have ready his instruments of all shapes and many in number so that he can extract bones if necessary and cut, elevate, saw, scrape, and grind them so as to attain a praiseworthy completion. Avicenna explains an instrument, a drill calling it trepanum or a perforator which does not go deeply into the interior of the surface of the bone. It is so called because it does not penetrate to the membrane since it has a round blunt extremity and a little ring which prevents the drill bit from going too deeply. Avicenna concurs in his chapter on skull fracture where he says: “When the necessity is verified for extending the wound and cutting into and extracting the bone then haste must be made and no delay in awaiting the completion of the pus in the wound should take place. This is to be understood particularly when the dura mater is compressed or punctured since such a puncture will cause an abscess or a spasm, perhaps leading to apoplexy. The bone should be extracted at once and sensation will return to the patient if apoplexy has set in “(19) .

Concerning the quality or properties of the medicine to be applied to the head after the operation, Avicenna advises the physician to lay a linen cloth soaked with rose oil over the mouth of the wound. He then continues urging the practitioner to take another cloth folded in two or three folds, soak it in wine and rose oil and smear the entire wound with rose oil. Ibn Sina emphasizes on application of rose oil because he believes that it is better to begin after laying bare of the bone in all wounds penetrating the cranium with a medicine which reduces pain, that is, with rose oil. Rather than with a medicine which is quite drying in its effects in order first to obviate symptoms such as an abscess and to preserve the complexion of the pstient. Secondly since in all wounds of the head and immediately after the bone is moved the humors begin to run, especially the hot ones, to the site of the wound therefore one must proceed with means which are cooling in their effects and somewhat styptic, such as rose oil.

## 4. Discussion 

Just beginning with the chapter on cranium fractures, Book IV, Avicenna puts much emphasis on this point that occasionally it happens that the cranium is fractured, but the skin is not ruptured and only creates abscess. So, he believes that if in this condition the physician deals with the abscess and the fracture is neglected, the bone beneath may be corrupted. Thus, Ibn Sina insists that whenever a fracture occurred in the cranium curing the abscess merely is not reasonable and it’s up to the practitioner to cut the skin to find the fracture and treat it as well. Avicenna in his chapter on skull fracture puts special emphasis on the importance of head injuries saying: “concerning those things which are harmful and cause it to be necessary is the fact that from other bones than the bones of the head the bandage drives back the pus and cannot be placed on the head. Thus, it is necessary to accept the bone and the fracture as it is in which there is a quantity of pus until it goes out of it. Likewise, if the poison happens to lie between the bone and the bandage pressing down on it the poison already generated will penetrate from the place to the marrow. We would then have to open and cleanse the wound in whatever member other than the head. There is all the more reason for completing the process in a head wound.” (20)

Note that Haly agrees on the timing with Paul in Practica IX, chapter on skull fracture. He says: “If the membrane is separated and the wound occurs in the patient in the winter the parts of bones must be removed entirely before the fourteenth day. If it happens in the summer then before the seventh day before the symptoms we mention occur.” Abulcasis agrees with these authorities in his Chirurgia III, chapter 3. Avicenna, however, seems to disagree with them concerning the time when he says: “There should be no delay in summer beyond the seventh day, in winter beyond the tenth, the sooner the better to avoid great harm.”

This difference of opinion among the authors mentioned in hastening the operation is readily recognized by a good physician. Note that other factors beside those mentioned are involved such as the time of year or season, age of the patient, region, complexion, his condition of humors, and such like. This is why Avicenna said the tenth, not the fourteenth day because his own region was very hot.

This evidences along with numerous unmentioned clues demonstrate that though Ibn Sina provided extremely systematic knowledge on head traumas along with both his observations and experiences and citations from the writing of the ancient physicians such as Galen and Paul of Aegina.As Aciduman puts it (21), however, Avicenna manages the patients not as a mere imitator, but an innovater and talented teacher of medicine and surgery.

## References

[1] Kshettry VR, Mindea SA, Batjer HH (2007). The management of cranial injuries in antiquity and beyond.. Neurosurgical Focus.

[2] Geddes J (2003). Trepanation: History, Discovery, Theory.. JRSM.

[3] Geddes J (2003). Trepanation: History, Discovery, Theory.. JRSM.

[4] Geddes J (2003). Trepanation: History, Discovery, Theory.. JRSM.

[5] GALEN JI (2006). Galen: on diseases and symptoms..

[6] Zimmerman LM, Veith I (1961). Great ideas in the history of surgery.

[7] Castioglioni A (1941). A history of medicine.. The American Journal of the Medical Sciences.

[8] Ackerknecht EH (1982). A short history of medicine..

[9] Loudon I (1997). Western medicine: an illustrated history..

[10] Sina I (1997). The canon of medicine..

[11] Sina I (2010). The canon of medicine..

[12] Sina I (2010). The canon of medicine..

[13] Sina I (2010). The canon of medicine..

[14] Sina I (2010). The canon of medicine..

[15] Sina I (2010). The canon of medicine..

[16] Sina I (2010). The canon of medicine..

[17] Sina I (2010). The canon of medicine..

[18] da Carpi JB (1990). On fracture of the skull or cranium..

[19] Berengario ca Carpi J, Lind L (1990). On fracture of the skull or cranium..

[20] Lind LR, da Carpi B (1990). Berengario da Carpi on fracture of the skull or cranium.. Transactions of the American Philosophical Society.

[21] Aciduman A, Arda B, Ozakturk FG, Telatar UF (2009). What does Al-Qanun Fi Al-Tibb (the Canon of Medicine) say on head injuries?. Neurosurg Rev.

